# Expectations for nursing care in newborn units in Kenya: moving from implicit to explicit standards

**DOI:** 10.1136/bmjgh-2017-000645

**Published:** 2018-03-21

**Authors:** Georgina A V Murphy, Gregory B Omondi, David Gathara, Nancy Abuya, Jacintah Mwachiro, Rose Kuria, Edna Tallam-Kimaiyo, Mike English

**Affiliations:** 1 Centre for Tropical Medicine and Global Health, Nuffield Department of Medicine, University of Oxford, Oxford, UK; 2 Health Services Unit, KEMRI – Wellcome Trust Research Programme, Nairobi, Kenya; 3 Nairobi City County Government, Nairobi, Kenya; 4 Ministry of Health, Nairobi, Kenya; 5 Nursing Council of Kenya, Nairobi, Kenya

**Keywords:** neonatal, newborn health, sick newborns, nursing, nurses, standards, guidelines, recommendation, hospitals, facility-based care, inpatient care, Kenya, Africa

## Abstract

Neonatal mortality currently accounts for 45% of all child mortality in Kenya, standing at 22 per 1000 live births. Access to basic but high quality inpatient neonatal services for small and sick newborns will be key in reducing neonatal mortality. Neonatal inpatient care is reliant on nursing care, yet explicit nursing standards for such care do not currently exist in Kenya. We reviewed the Nursing Council of Kenya ‘Manual of Clinical Procedures’ to identify tasks relevant for the care of inpatient neonates. An expert advisory group comprising major stakeholders, policy-makers, trainers, and frontline health-workers was invited to a workshop with the purpose of defining tasks for which nurses are responsible and the minimum standard with which these tasks should be delivered to inpatient neonates in Kenyan hospitals. Despite differences in opinions at the beginning of the process, consensus was reached on the minimum standards of neonatal nursing. The key outcome was a comprehensive list and grouping of neonatal nursing task and the minimum frequency with which these tasks should be performed. Second, a simple categorisation of neonatal patients based on care needs was agreed. In addition, acceptable forms of task sharing with other cadres and the patient’s family for the neonatal nursing tasks were agreed and described. The process was found to be acceptable to policy-makers and practitioners, who recognised the value of standards in neonatal nursing to improve the quality of neonatal inpatient care. Such standards could form the basis for audit and quality evaluation.

**Key questions**

**What is already known about this topic?**

- Access to essential inpatient services with adequate nursing care for small and sick newborns will be key if progress is to be made in reducing neonatal mortality in low- and middle-income countries.
- Although the performance of key nursing tasks is described in detail in nursing manuals, there are currently no agreed written standards to guide the delivery of nursing care for neonatal patients in Kenya.
- Instead, standards are implicitly defined as historical practice norms that vary, often quite markedly, from one facility to the next.

**What are the new findings?**

- An expert group comprising major stakeholder organisations, policy-makers, nursing trainers, and frontline workers spanning private and public sectors can work together, respecting the challenges of varying contexts, to reach consensus on defining minimum standards of neonatal nursing care.
- Levels of dependency of neonatal patients were defined with the recognition that different patients may require different standards of care depending on the severity of their condition and dependency on nursing care.

**Key questions**

**Recommendations for policy**

- Minimum standards of neonatal nursing care could be used as benchmarks for quality evaluation across private and public sector facilities.
- Highly practical operational research priorities were identified, these included the need to identify/design more structured nursing notes (cardex) and better tools to document nursing observations to facilitate more rapid, accurate, and informative documentation and communication between nurses and with other professionals as part of improving quality of care.
- Clearly defining expectations of neonatal nursing care may support discussions on the role of different health worker cadre in providing neonatal care, task-shifting policy, neonatal nursing training needs, and setting appropriate nurse to patient ratios.

## Introduction

The need to reduce neonatal mortality has become a priority internationally, with the Sustainable Development Goal (SDG) three targeting to reduce mortality to 12/1000 live births or lower.^1^ In Kenya, substantial efforts will be needed to reduce the current neonatal mortality of 22/1000 live births to reach this SDG target by 2030. Access to basic but high quality inpatient neonatal services for small and sick newborns will be key if progress is to be made.^2 3^ Sick newborns require continual supportive care and observation, with repeated and often multiple interventions delivered 24 hours a day, and their condition can change rapidly. Good outcomes for this patient population are, therefore, particularly dependent on nursing care.

The WHO has helped lead efforts to set standards for neonatal care, including recent indicators on all aspects of peripartum care.^4^ A number of these indicators touch on quality of the nursing process; many of these implicitly require high quality nursing care provision. High income countries, such as the UK have produced valuable detailed guidance on neonatal nursing.^5 6^ Equivalent standards do not currently exist in many low-resourced setting, including Kenya.

To complement these ongoing international efforts to define standards for and indicators of care for small and sick newborns, we set out to consider specifically defining minimum standards of neonatal nursing care in Kenya. In doing this, we aimed to initiate a discussion on explicit nursing care standards. We aimed to provide locally agreed benchmarks that can support evaluations of the quality of inpatient neonatal nursing care.

## Exploring standards of neonatal nursing in Kenya

### The setting

Nursing care within the newborn unit is predominantly provided by registered nurses. Diploma and degree level registered nurses receive 2 and 4 weeks of training in newborn care, respectively. Since 2012, a specialist 1 year post-basic training in neonatal nursing was approved by the Nursing Council of Kenya; however, the number of nurses trained so far at national level is only approximately 100 nurses. Thus, the vast majority of nurses providing neonatal care in Kenyan hospitals learn their skills during practice, where there are no nurses with specialist neonatal nursing qualifications. Nurse training and practice in Kenya is regulated by the Nursing Council of Kenya (NCK). The National Nurses Association of Kenya is a national professional association representing nurses’ views and interests, with sub-chapters for nursing specialities.

Our particular focus was inpatient care for small and sick newborns. This is typically delivered through a newborn unit that aims to provide a minimum package of essential interventions, for example: feeding support with nasogastric tubes and intravenous fluids; infection prevention and management, including antibiotics; oxygen provision and (less often) continuous positive airway pressure (CPAP); and phototherapy for jaundice.^3 4^ The role of nurses in the delivery of such care is central.^7^ However, there are extreme staff shortages in many facilities in Kenya.^8–10^ Neonatal patient to nurse ratios as high as 15:1 have been previously described. Such ratios are in extreme contrast to the internationally recommended ratios of newborns to nurses of: 4:1 for basic or continuing care; 2-3:1 for stable babies requiring intervention; and 1:1 for high-dependency care.^11^ Quality of care for neonatal patients varies widely between health facilities and across sectors (private and public) in terms of their structural capacity to provide care and indicators of the process of care. Although, indicators capturing key nursing processes are typically lacking.

### Identifying key neonatal nursing tasks

We began by trying to identify nursing standards documents in Kenya by reaching out to experts, the Ministry of Health, and the Nursing Council of Kenya (NCK). The only document that emerged as being relevant was the NCK ‘Manual of Clinical Procedures’.^12^ The manual applies to all areas of nursing and is a key reference text for nurse training institutions in Kenya. While the manual offers detailed standard operating procedures on many nursing procedures, it does not offer guidance on how often tasks should be done nor by whom. Instead, such operational decisions are made at each facility and are often implicitly rather than explicitly defined. We are not aware of the availability of such neonatal nursing standards from any sub-Saharan African countries.

Drawing on this manual, medical and nursing team members identified tasks relevant for inpatient neonatal care and organised these into domains (eg, vital signs and monitoring, oxygen treatment, etc). We sought to present these to an expert advisory group for more detailed consideration.

### Expert advisory group

We constituted an expert advisory group (n=12; see Nursing Tasks Advisory Group in the author list below) made up of individuals responsible for: delivery of neonatal care in major public and private hospitals; neonatal nurse training; and child health and nursing services policy in the Ministry of Health and a County Government. This group also included major nursing stakeholder groups, the National Nurses Association of Kenya and NCK. These members of the expert advisory group were selected based on their first-hand experience of providing neonatal nursing care, neonatal medical care, training nurses, and designing and implementing nursing policy in Kenya.

The group was invited to a daylong workshop with the purpose of defining (1) what tasks are regarded as the responsibility of nurses providing inpatient neonatal care and (2) the minimum frequency of tasks (where relevant). We aimed to move from implicit knowledge, where nurses are socialised into ‘how to do’ things on the wards and have an implicit understanding of what is an acceptable minimum standard of care, to making this expert knowledge more explicit. In doing so, we also aimed to explore whether we could achieve consensus on explicit minimum standards that could be useful in improving quality of care going forward.

The expert advisory group began by reviewing the compiled list of neonatal nursing tasks. Missing tasks were added and tasks deemed to be outside of the remit of nurses were removed. Four groups were formed with a facilitator supporting each group. Each group focused on a subset of tasks (domains). Employing a nominal group strategy, each group discussed i) the minimum frequency with which these tasks should be done, ii) who else might reasonably do these tasks, and iii) how operationally sensible it was to ‘bundle’ tasks during care delivery. Once within-group consensus was reached, cross-group consensus was sought through discussion across all expert advisors. Facilitation was provided by a senior researcher, with consensus positions reached by show of hands.

## Recommendations of the expert advisory group

The final standards proposed are described in table 1. All of the tasks listed were deemed currently done by nurses. However, it was noted that some of these tasks (such as preparing feeds) would not be the responsibility of nurses in a well-resourced environment. These tasks should instead be done by specialised staff, such as nutritionists or clinicians (either a non-specialist physician or a clinical officer (non-physician clinician) with specialist training in paediatrics), as relevant. Additionally, some tasks were considered best carried out by the patient’s family (eg, cleaning/bathing/clothing of baby) or support staff (eg, cot cleaning) but overseeing these tasks was endorsed as remaining the responsibility of the nursing team. It should be noted that developmental care and positioning tasks were not highlighted or discussed by the expert advisory group. These tasks, traditionally done by a physiotherapist or nurse, are important for the long-term healthy development of neonates. The absence of these tasks from the recommendations may have been due to the framing of the working group discussions or may reflect an apparent lack of prioritisation of these roles in Kenya at the moment.

**Table 1:** Neonatal nursing tasks

Task Area	Task done by	Frequency (minimum daily requirement)	Comment
**Admission**			
Admission nursing history, clinical evaluation and vital signs	Nurse	On admission	
**Routine vital signs and monitoring**			
Temperature (including incubator temperature)	Nurse	*Normal:* four times daily/6 hourly *Category C patients:* two times daily/12 hourly	KMC newborns: Vital signs monitoring and monitoring of general clinical condition
Pulse	Nurse		
Respiration	Nurse		
Checking and documenting oxygen saturation for babies not on oxygen (for babies on oxygen see section below).	Nurse	Four times daily/6 hourly	Although it is recognised that the availability of pulse oximeters is limited at present, checking oxygen saturation in sick babies not on oxygen should be promoted.
Skin colour	Nurse	*Normal:* four times daily/6 hourly *Category C patients:* two times daily/12 hourly	Conduct together with vital signs monitoring
Jaundice	Nurse		
Respiratory effort	Nurse		
Abdominal distension	Nurse		
Weight	Nurse/clinician	Alternate days	
Input/output - general	Nurse	Four times daily/6 hourly	
Input - IV fluids	Nurse/clinician	Frequency depends on prescription. Infusion rate checked and documented three hourly.	Conduct together with vital signs
Input/output documentation (amount that has been infused)	Nurse	Three hourly	
**Regular patient checks/care**			
Changing diapers/checking for stool and urine	Nurse/patient’s family	As required	Done with vital signs and document passing stool and urine during diaper change
Cleaning/bathing/clothing	Patient’s family	As required	
Changing bed linens	Nurse/patient’s family	As required	
Incubator monitoring and settings	Nurse	During shift changes/per shift	
Wound care (checking/renewing dressings)	Nurse	As required	
**Administering interventions/doing investigations**			
Taking venous blood	Nurse/clinician	As required	Nurses can perform this task if they have the skills to do so and if the clinicians charged with the responsibility are unavailable.
Taking heel-prick blood	Nurse/clinician	As required	
Collecting urine/stool	Nurse/patient’s family	As required	
Resuscitation with bag valve mask	Multidisciplinary	As required	
**Drugs and vaccines**			
Drug preparation	Nurse	As per drug schedule	
Dilutions (compatibility)	Nurse		
Oral drug administration	Nurse		
IV drug Administration	Nurse		
Cannula patency check	Nurse	Before IV drug administration	Test if line is patent with water for injection
Checking cannula sites	Nurse	During shift changes/twice a day	Visual inspection and palpation of the soft tissue for localised infection
Giving vitamin K	Nurse	At birth/as required	
Routine cord care - antiseptic application	Nurse/patient’s family	Once daily	
Eye care - routine drops application	Nurse/patient’s family	Once daily	
OPV vaccination	Nurse	As required	
BCG vaccination	Nurse	As required	
**Oxygen**			
Checking tube position and nostril care/damage	Nurse	Eight times daily/3 hourly	
Initiating and regulating oxygen flow	Nurse	As required	
Documenting oxygen treatment	Nurse/clinician	As required	
Checking and documenting pulse oximetry	Nurse/clinician	Three hourly/as required	For patients on oxygen
Monitoring/regulating pressure	Nurse/clinician	Three hourly/as required	Regulating pressure is done by clinician
Checking nose/cleaning airway	Nurse	Three hourly/as required	
Checking respiration	Nurse	Three hourly/as required	
Checking and changing humidifier	Nurse	As required	
**CPAP management**			
CPAP machine setup	Nurse/clinician	As required	
Applying nasal prongs/fixing tubing	Nurse/clinician	As required	
**Phototherapy**			
Checking eyes for damage	Patient’s family under supervision by nurse	Four times daily/6 hourly	
Skin colour	Nurse/family	Four times daily/6 hourly	Conduct together with vital signs
Checking exposure/baby positioning	Shared by clinical team	Continuous/6 hourly/per shift	
Fixing eye pad	Patient’s family under supervision by nurse	Continuous/6 hourly	
Documenting phototherapy	Nurse/clinical team	Shift change/continuous	Done during admissions and as required
**Expressed breastmilk and formula milk preparation**			
Formula making	Nurse/nutritionist/mother	Eight times daily/3 hourly	
Storage and labelling of expressed breastmilk	Nurse/nutritionist/mother	Eight times daily/3 hourly	
Measuring volumes for individual patients	Nutritionist	Continuous	
Disinfection of cups	Nurse/patient attendant	Eight times daily/3 hourly (after every feed)	
**Feeding**			
Teaching/counselling on breastfeeding (attachment/suck)	Nurse/nutritionist	On admission and as required/daily	
Checking feed prescribed/type of feed	Nurse/mother under supervision	Three hourly or as per feeding schedule	
Cup feeding	Nurse/mother under supervision	Eight times daily/3 hourly	
Nasogastric tube feeding/checking nostril	Nurse/mother under supervision	Eight times daily/3 hourly	Nostril should be checked by a nurse when administering drugs
Checking residual gastric volumes (nasogastric aspiration)	Nurse/mother under supervision	Eight times daily/3 hourly	
Charting feed volumes/times	Nurse/mother under supervision	Eight times daily/3 hourly	
Nasogastric tube insertion	Nurse	As required and replace after every 3 days	
**Blood transfusion/exchange transfusion**			
Cross-checking blood for transfusion with co-worker	Nurse	As required	
Transfusion chart (patient observations/volume of blood)	Nurse	1/4 hourly	
Pre-administration check of laboratory results/medical record	Clinician	As required	
Exchange transfusion progress	Nurse/clinical team	Continuous with clinical team during procedure	
**Documentation**			
Discharge and admission registration	Nurse	As required	Nurses would benefit from clerical assistants, but nurses are responsible.
Patient labels	Nurse	As required	
Notifications – Birth	Nurse	As required	
Notifications – Death	Clinician/HRIO	As required	
Treatment sheets review	Nurse/clinician	Once daily	
Incident book	Nurse	As required	
Updating mother/child health book (vaccines, weight etc.)	Nurse	As required	
Recording in drug books	Nurse	As required	
Billing	Multidisciplinary	As required	Services free in public hospitals, therefore no billing
Recording of stocks – non-pharmaceuticals	Nurse	As required	
Managing medical records	Nurse/clinical team	Continuous/as required	
**Counselling/support**			
Parent - counselling, answering questions about clinical/nursing care	Nurse	Continuous/as required	
Support for KMC	Shared with clinician and patient’s family	As required	
Supervision of mother during KMC	Nurse/clinician	As required	Experienced mothers could assist in helping the other mothers
Expressing breastmilk	Nurse/ nutritionist/ experienced mothers	As required	
Health education and progress	Nurse	As required	
Post-discharge care advice	Nurse	On discharge/as required	
Instructions for drugs/medication on discharge	Nurse/pharmacist	On discharge	Nurse reinforces medication instructions for post-discharge use during the discharge process after or before patient obtains medication from the pharmacy, depending on hospital policy.
Family planning	Nurse	As required	
HIV/STI prevention	Nurse/counsellor/clinician	As required	
Bereavement counselling	Nurse/clinician	As required	
**Infection control and cleaning**			
Cot cleaning	Support staff	Daily cleaning and thorough cleaning after discharge of a baby before another uses it and as required	
Cleaning incubator	Support staff		
Hand washing	Multidisciplinary	As required	
Visitors education/practice (on gowns/shoes/hand hygiene)	Nurse	As required	
**Miscellaneous**			
Providing input to medical ward rounds	Nurse	During ward rounds	
Accompany to lab/X-ray/theatre for procedure or operation	Nurse	As required	
Accompany on outward referral to another facility	Nurse	As required	
Last offices (stopping interventions and preparing documentation after death).	Nurse	As required	Washing the body and anything else should be done by mortuary or support staff.
Pre-operative and post-operative care	Nurse	As required	
Assistance with portable chest X-ray	Nurse/radiology team	As required	
Preoperative and postoperative care	Nurse	As required	
Setting alarms (incubator)	Nurse	As required	Part of 6 hourly review and checks
Equipment checks	Nurse/biomedical team	As per schedule/as required	
Equipment handover	Nurse	Once per shift	

Tasks listed as being done by nurses can also be done by students under supervision of a qualified nurse, who is responsible for confirming that the task has been done correctly and as per hospital policy. However, students are not to carry out any tasks for category A patients. Clinician refers to either a generally trained (non-specialist) physician or a clinical officer (non- physician clinician) with specialist training in paediatrics.

HRIO, health records and information officer; IV, intravenous; KMC, kangaroo mother care; STI, sexually transmitted infection; OPV, oral polio vaccine; BCG, Bacillus Calmette–Guérin.

Supervision of students was also discussed, as they carry out some of the tasks. The range of tasks delegated varied by institution and type of student. Yet, there was agreement that it should be an explicit standard that: if a qualified nurse staff delegates a task to a student or other carer, the nurse retains responsibility for ensuring such tasks are performed correctly and safely, including providing appropriate supervision.

Defining level of dependency of neonatal inpatients, for which there were previously no widely agreed criteria, was recognised as essential to defining standards for the frequency of performance of nursing tasks. Recommendations on levels of dependency focused on facilities typical of the public sector, with proposed simple categories described in box 1. This categorisation reflects the lack of neonatal intensive care in most Kenyan hospitals; a fourth category that encompasses expensive and complex neonatal intensive care provided in high income setting might be relevant in some settings, such as large costly private sector facilities. Further work is needed to more clearly and precisely define levels of neonatal care in Kenya. In doing so, these categories of dependency should also be refined.

Box 1 **Categorisation of neonatal patients**

**Category A babies**

- Babies on oxygen/continuous positive airway pressure (CPAP) or intravenous fluids, who are acutely ill and unstable and require the closest monitoring and a higher level of care. For such babies, delegation of tasks to students or others would need to be done only under very close supervision.

**Category B babies**

- Babies who have stabilised but may still be ill and receiving, for example, assisted feeding (e.g. nasogastric feeds) and intravenous drugs or who require close monitoring, for example a baby on double phototherapy, with intermittent convulsions or at risk of apnoea.

**Category C babies**

- Babies who are stable requiring only monitoring or oral medications often after stepping down from category A or B care. Many of these should be receiving kangaroo mother care (KMC) or, for example, may be stable abandoned babies or term babies requiring phototherapy only or accommodation because of severe maternal illness. These babies may require regular feeding and changing – done by parents wherever possible – but limited care in terms of nursing observations.

## Reflections of the expert advisory group

At the beginning of the discussion there were disagreements between experts on the appropriate standards of care. Policy-makers or representatives of professional bodies tended to propose higher, more aspirational standards. Front-line workers argued that these ignored the reality of providing care. Differences in opinion were also expressed between health-workers providing care in public versus private sector facilities due to differences in resources. For example, experts working in the private, high-resource sector suggested observations in higher dependency babies should be done 4 hourly. Though, they accepted that this was only realistic in a small number of private facilities. Accepting the need for minimum standards, the group was able to reach consensus spanning all parties.

A number of discussion points that highlighted challenges faced by nurses in neonatal care were raised during the process. These challenges might be helped by disseminating agreed standards. It was recognised by the group that nurses often take on multiple additional roles, which should ideally be done by nutritionists, clinicians, and/or radiologists, in the interest of the care and well-being of the baby. For example, nurses may need to prepare feeds or take blood specimens because of shortages of other professional staff. Recognising this *de facto* task sharing, and to protect nurses legally, recent policy changes have expanded the role of nurses through the Kenyan Task Sharing Policy Guidelines 2017–2030.^13^

The formal standards of care proposed were seen by the expert group as a way to improve the professionalism within neonatal nursing. It was also noted that such standards may be a helpful means to lobby managers and governments for the resources and personnel necessary to achieve minimum standards of care. Overall, the process of creating these recommendations was found to be acceptable to policy-makers and practitioners. They recognised the value of standards as a means to improve the quality of neonatal inpatient care in Kenya. The expert group also felt that it was reasonable to evaluate service delivery against the recommended standards.

A number of important issues that would benefit from local operational research also arose. These particularly concerned the importance of documentation of nursing care, which can occupy a considerable proportion of nursing time. It was recognised that documentation was often fragmented, rather than systematic. Experts suggested that there would be benefits from developing more structured nursing notes with better tools for recording nursing observations. It was felt that these might improve communication between nurses and with other professionals, and reduce the time burden of unnecessary and unhelpful documentation, with the potential to considerably improve the quality of care.

## Conclusion

There is currently a distinct lack of detailed practical guidance for neonatal nursing standards in many resource-limited settings. In this paper, we describe a process of working with experts to develop locally relevant and acceptable recommendations for minimum standards of neonatal nursing care in Kenya. We hope that our efforts to more clearly define standards help health-workers and policy-makers to operationalise their aspirations for better care. Moving standards from being part of location specific, tacit knowledge to becoming commonly accepted and explicit provides some basis for developing credible audit criteria. Such criteria may help ensure that all facilities, across the public and private sectors, provide inpatient neonatal care services to at least a minimum standard of care. Furthermore, such standards may be helpful for discussions on health-worker shortages, including defining the role of nurses in neonatal care,^14 15^ exploring task-sharing and task-shifting policy,^13 16^ understanding nursing training needs,^17^ and setting appropriate nurse to patient ratios.^11 18^ An agreement on minimum standards creates a baseline for discussion on how to both clearly define the role of nurses and improve neonatal care in this low-resource, high-burden setting. Such standards also form a platform for further discussions to refine definitions of levels of care and to develop wider guidance spanning the multi-professional nature of neonatal care.
